# A clinical case of typical anorexia nervosa in a prepubescent boy

**DOI:** 10.1192/j.eurpsy.2021.956

**Published:** 2021-08-13

**Authors:** A. Bryukhin, M. Sologub, E. Sineutskaya, M. Matsneva, A. Zakharova, E. Okonishnikova, T. Lineva

**Affiliations:** 1 Department Of Psychiatry And Medical Psychology, RUDN University Moscow., Moscow, Russian Federation; 2 Medical Clinic, Center for ED research in Moscow - CIRPP, Moscow, Russian Federation

**Keywords:** eating disorder, anorexia nervosa

## Abstract

**Introduction:**

Clinical case of 10-year-old patient with anorexia nervosa at the stage of severe cachexia. Features of the disease, diagnosis, treatment and methods of restoration of nutrition. Anorexia nervosa is an eating disorder (ED), severe pathology, manifested by severe complications, high disability and can cause death. ED has traditionally been perceived as a disease affecting women. However, this pathology occurs men, is 10-25% of the total number of patients with ED, or 1-2% in the population.

**Objectives:**

Describe the difficulties in identifying ED in men that affect diagnosis and treatment, especially if they are underage patients.

**Methods:**

Patient I. 10 years, selectivity in food from 4 years, during the week before hospitalization complete rejection of food and water. When entering the clinic height 127, weight 19 kg, BMI 11. In the clinical picture anxiety, low mood, fear of eating and weight gain. The duration of the disease for about one year. Clinical and psychopathological method.

**Results:**

Diagnosis F50.0 anorexia nervosa in the stage of severe cachexia. Treatment: olanzapine and fluvoxamine in the age dosages, parenteral Kabiven infusion, individual and group psychotherapy. Psychoeducation of parents and Maudsley method therapy. As a result of treatment improved mood, decreased anxiety associated with eating and weight gain. At the time of discharge from the clinic height 127, weight 30 kg, BMI 18.

**Conclusions:**

The clinical case indicates the need to increase the attention of pediatricians, psychologists, psychiatrists and other doctors in connection with the growth and rejuvenation of ED in the male population.

